# Accelerated phase Ia/b evaluation of the malaria vaccine candidate PfAMA1 DiCo demonstrates broadening of humoral immune responses

**DOI:** 10.1038/s41541-021-00319-2

**Published:** 2021-04-14

**Authors:** Edmond J. Remarque, Bart W. Faber, Roberto Rodriguez Garcia, Herman Oostermeijer, Sodiomon B. Sirima, Issa Nebie Ouedraogo, Leila Kara, Odile Launay, Sophie Houard, Odile Leroy, Clemens H. M. Kocken

**Affiliations:** 1 Biomedical Primate Research Centre, Rijswijk, The Netherlands; 2 Centre National de Recherche et de Formation sur le Paludisme (CNRFP), Ouagadougou, Burkina Faso; 3 Assistance Publique-Hôpitaux de Paris (AP-HP), Hôpital Cochin, CIC Cochin-Pasteur, Paris, France; 4 European vaccine initiative, Heidelberg, Germany; 5 Present Address: Department of Virology, Biomedical Primate Research Centre, Rijswijk, The Netherlands; 6 Present Address: Groupe de Recherche Action en Santé (GRAS), Ouagadougou, Burkina Faso

**Keywords:** Malaria, Protein vaccines, Malaria

## Abstract

*Plasmodium falciparum* apical membrane antigen 1 (PfAMA1) is a candidate malaria vaccine antigen expressed on merozoites and sporozoites. PfAMA1’s polymorphic nature impacts vaccine-induced protection. To address polymorphism, three Diversity Covering (DiCo) protein sequences were designed and tested in a staggered phase Ia/b trial. A cohort of malaria-naive adults received PfAMA1-DiCo adjuvanted with Alhydrogel® or GLA-SE and a cohort of malaria-exposed adults received placebo or GLA-SE adjuvanted PfAMA1 DiCo at weeks 0, 4 and 26. IgG and GIA levels measured 4 weeks after the third vaccination are similar in malaria-naive volunteers and placebo-immunised malaria-exposed adults, and have a similar breadth. Vaccination of malaria-exposed adults results in significant antibody level increases to the DiCo variants, but not to naturally occurring PfAMA1 variants. Moreover, GIA levels do not increase following vaccination. Future research will need to focus on stronger adjuvants and/or adapted vaccination regimens, to induce potentially protective responses in the target group of the vaccine.

## Introduction

Intensive efforts have led to a reduction of the malaria burden. Unfortunately, it remains a major cause of morbidity and mortality, especially in children in sub-Saharan Africa. Effective vaccines would be a valuable addition to the arsenal needed to further reduce the impact of malaria. Currently only one moderately efficacious malaria vaccine (RTS,S), addressing the pre-erythrocytic stage, is implemented in three national (pilot) programmes^1^.

A strategy to improve malaria vaccine efficacy would be the inclusion of additional blood-stage antigens, of which a number are currently in clinical development (e.g., Rh5, CyRPA, RIPR, MSP3, GLURP, SERA5, EBA & AMA1)^2^. One of these blood-stage candidates is the ectodomain of *Plasmodium falciparum* Apical Membrane Antigen 1 (AMA1)^3,4^. AMA1 plays an essential role in the red blood cell invasion cascade^3,5–7^ and anti-AMA1 IgG inhibits red blood cell invasion^8,9^. It is also expressed on sporozoites and may thus also confer protection against hepatocyte invasion^10^. However, it is fair to state that the expectations based on the above observations are not substantiated by clinical phase II studies^11^, nor by vaccination-challenge studies using the Controlled Human Malaria Infection model^12^. However, PfAMA1 could still be considered as a candidate as part of a multi-stage, multi-component malaria vaccine. The ultimate target population for such a vaccine would be infants in malaria-endemic countries.

PfAMA1 shows considerable sequence diversity; with a PfAMA1 database currently at 4289 entries (Pubmed Nucleotide, last accessed April 24, 2020), 167 positions out of 622 are polymorphic and at least 1383 unique sequences were found [Remarque, unpublished]. The 449 amino acids (aa) long ectodomain of PfAMA1 (DI, DII and DIII) has 142 polymorphic positions with a maximum between sequence difference of 42 aa. Many of these variants can be found simultaneously at a single study site^13^. Owing to this sequence diversity, about half of the IgG response induced following vaccination cross-reacts functionally with heterologous variants^14^. The three PfAMA1 Diversity Covering (DiCo) proteins have been designed to, in combination, cover naturally occurring AMA1 amino acid polymorphisms^4^. Vaccination of rabbits and monkeys with a mixture of three DiCo proteins yielded broadly cross-reactive antibodies capable of inhibiting the in vitro growth of several laboratory malaria strains^4,15^. This broadening of the antibody response was shown to be due to an increased amount of cross-reactive antibodies, likely due to the dilution of strain-specific epitopes in the three vaccine antigens^16–19^.

The phase I fast-track strategy, designed by the European Vaccine Initiative (www.euvaccine.eu), is a first-in-human evaluation done in a staggered multi-centre phase Ia/b clinical trial involving both malaria-naive and malaria-exposed adults and thus allows direct comparison of anti-AMA1 immune responses in both populations^20^.

Here, we present data obtained from an in-depth analysis of immune responses following PfAMA1-DiCo vaccination comparing anti-PfAMA1 immune responses in malaria-naive and malaria-exposed adult volunteers. These data facilitate the comparison of the breadth of the response generated by a vaccine designed to induce broadly cross-reactive responses in naive adults to the breadth of the anti-AMA1 response naturally acquired by repeated parasite exposure in semi-immune adults.

## Results

### IgG levels to 7 AMA1 variants in malaria-naive adults

**Fig. 1:**
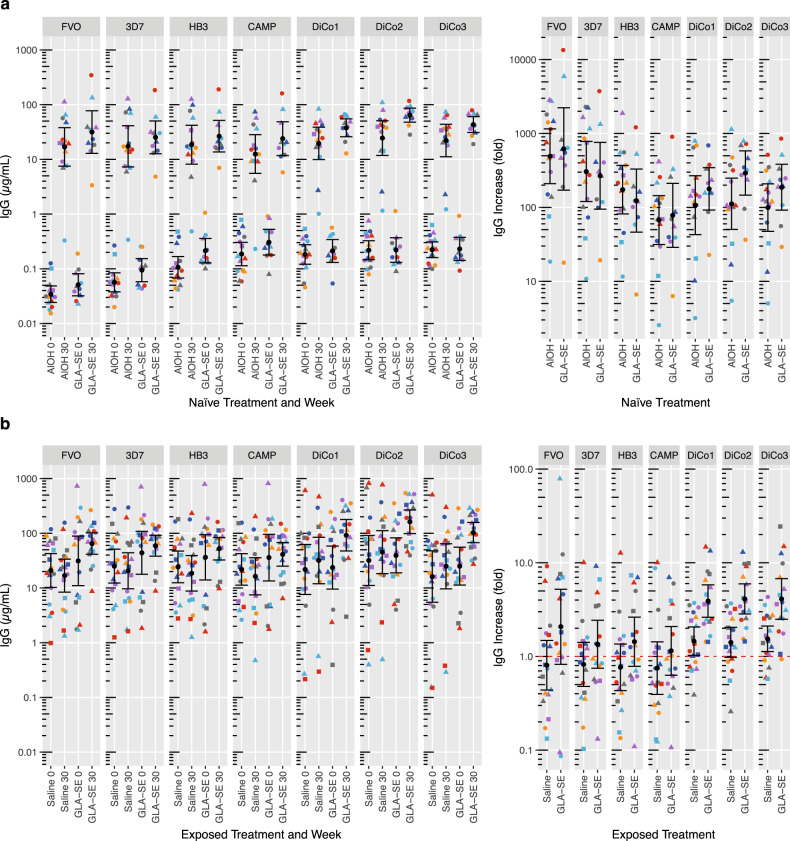
IgG levels to 4 natural AMA1 variants and the DiCo variants before and 4 weeks after the third vaccination. **a** Malaria-naive and **b** malaria-exposed adults. Individual IgG levels before and four weeks after the third vaccination and IgG level ratios between week 30 and week 0 are shown together with the IgG fold-increase. Colours and shapes within treatment groups indicate individual subjects. Summary statistics (geometric means with 95% confidence intervals) are shown in every panel. The red dashed line in the IgG increase indicates no change in IgG (ratio post/pre-vaccination = 1).

To measure the induced breadth of the antibody response, following AMA1 DiCo vaccination, we measured IgG levels in malaria-naive adults to four natural AMA1 variants and the DiCo variants at week zero and four weeks after the third vaccination (Fig. 1a). The number of amino acid differences between the seven AMA1 variants is shown in supplementary table 1. Before vaccination IgG levels ranging from 0.1 to 0.3 µg/mL were observed for the three DiCo, HB3 and CAMP variants, whereas pre-vaccination levels for the FVO and 3D7 variants were significantly lower (0.04 and 0.07 µg/mL, respectively (all *p* < 0.013 Tukey HSD)). Four weeks after the third vaccination IgG level rises ranging between 67 (CAMP Alhydrogel®) and 618-fold (FVO GLA-SE) were observed for the 7 AMA1 variants (Fig. 1a right-hand panel). Titre rises for FVO were significantly higher than those for DiCo1, DiCo3, HB3 and CAMP (4.1 [13 to 13.3], *p* = 0.009, 4.2 [1.3 to 13.6], *p* = 0.007, 3.6 [1.1 to 11.7], *p* = 0.025 and 7.6-fold [2.3 to 24.6], *p* < 0.00001, respectively). Titre rises for CAMP were significantly lower than those for 3D7 (0.25-fold [0.08 to 0.81], *p* = 0.01). IgG level rises tended to be higher for the 3 DiCo vaccine variants (twofold, 0.71 to 5.7) in the GLA-SE as compared to the Alhydrogel® group (*p* = 0.17, LMM), while by contrast rises for the 4 natural variants did not differ for the treatment groups (0.98-fold (0.29 to 3.3), *p* = 0.98, LMM). The anti-AMA1 IgG levels observed four weeks following the third vaccination ranged between 12.5 and 64.2 µg/mL for the CAMP Alhydrogel® and DiCo2 GLA-SE groups, respectively (Fig. 1a) and were approximately 1.4 to 2.6-fold higher in the GLA-SE group as compared to the Alhydrogel® group, albeit not statistically significant.

### Malaria infection incidence in malaria-exposed adults

Vaccinations in the Burkinabe volunteers started at the start of the transmission season, therefore data on the incidence of asymptomatic and symptomatic parasitaemias are provided (Supplementary Table 2). The incidence of the first parasitaemia episode (either asymptomatic or symptomatic) is shown in Supplementary Fig. 1 (panel A). A total of 29 first episodes were observed between week zero and 30, of which 16 occurred in the Saline- and 13 in the GLA-SE group. Four volunteers remained free of malaria (one in Saline and three in GLA-SE group, Supplementary Table 2B). The incidence of the first symptomatic malaria episode is shown in Supplementary Fig. 1 (panel B). A total of 12 symptomatic episodes were observed between week zero and 30, of which 5 were in the Saline- and 7 in the GLA-SE group (Supplementary Table 2C).

### IgG levels to 7 AMA1 variants in malaria-exposed adults

IgG levels in malaria-exposed adults to four natural AMA1 variants and the DiCo variants at week 0 and 4 weeks after the third vaccination are shown in Fig. 1b. Before vaccination IgG levels ranging between 20.0 and 35.4 µg/mL were observed for the seven AMA1 variants under investigation; no significant differences were observed between treatment groups in pre-vaccination levels for the seven AMA1 variants (*p* > 0.81).

Following vaccination IgG levels to the DiCo vaccine variants increased approximately fourfold in the GLA-SE group (3.9 [2.6 to 5.8], 4.1 [2.9 to 6.0] and 4.1 [2.5 to 6.8], for DiCo1, 2 and 3, respectively) and about 1.5-fold in the Saline group (1.5 [1.03 to 2.1], 1.4 [0.98 to 2.0] and 1.5 [1.1 to 2.1], for DiCo1, 2 and 3, respectively) (Fig. 1b). Fold-increases to the DiCo vaccine variants were 2.7-fold higher (1.7 to 4.5) in the GLA-SE group as compared to the placebo group (*p* = 0.0003, LMM). IgG levels to the four natural AMA1 variants increased slightly, but not significantly in the GLA-SE group (2.1 [0.82 to 5.2], 1.4 [0.5 to 2.6], 1.4 [0.7 to 2.4] and 1.2 [0.6 to 2.1] for FVO, HB3, 3D7 and CAMP, respectively) (Fig. 1b). In the Saline controls, IgG levels to the four natural AMA1 variants decreased slightly, but not significantly (0.8 [0.4 to 1.5], 0.8 [0.4 to 1.4], 0.8 [0.5 to 1.4] and 0.7 [0.4 to 1.4], for FVO, HB3, 3D7 and CAMP, respectively). Fold-increases to the natural AMA1-variants tended to be 1.9-fold (0.9 to 4-fold) higher in the GLA-SE as compared to the Alhydrogel® group, albeit not statistically significant (*p* = 0.11, LMM).

Post-vaccination Anti-AMA1 IgG levels in the Saline group ranged between 16.4 and 45.1 µg/mL for CAMP and DiCo2, respectively, whereas in the GLA-SE group levels ranged between 41.1 and 162.4 µg/mL for FVO and DiCo2, respectively. Post-vaccination IgG levels were between 2.5 and 4.1-fold higher in the GLA-SE as compared to the Saline group for CAMP and DiCo3, respectively; significantly higher IgG levels were observed in the GLA-SE as compared to the Saline group for FVO (3.9-fold [1.2 to 12]), DiCo2 (3.6-fold [1.1 to 12]) and DiCo3 (4.1-fold [1.3 to 13]). Supplementary Fig. 2 (panel A) shows that there were no IgG fold-increase differences within the treatment groups when subjects with or without a clinical malaria episode are compared.

### DiCo vaccination-induced IgG breadth in malaria-naive adults is similar to IgG breadth in placebo-immunised malaria-exposed adults

To confirm that AMA1-DiCo vaccination induces a broad response against AMA1 variants, we compared IgG levels to several AMA1 variants in vaccinated malaria-naive to those in malaria-exposed adults receiving placebo or GLA-SE adjuvanted AMA1-DiCo. Week 30 IgG levels to 7 AMA1 variants in vaccinated naive volunteers were not significantly different from those in placebo-vaccinated Burkinabe as demonstrated with week 30 naive/exposed IgG ratios ranging between 0.54 and 1.88 (Table 1). Comparison of week 30 IgG levels shows that geometric means in the French Alhydrogel® group are lower (between 0.15 and 0.36-fold) than those in AMA1-DiCo GLA-SE vaccinated exposed subjects, with significantly higher IgG levels in the exposed subjects for FVO, 3D7, DiCo1, DiCo2 and DiCo3 (Table 1). Week 30 IgG levels in the naive GLA-SE group compared to those in the Burkinabe GLA-SE group shows that geometric means in the naive group are lower than those in the exposed group (between 0.4 and 0.58-fold), albeit without achieving statistical significance.

**Table 1:** Comparison of Week 30 IgG levels in malaria-naive and malaria-exposed adults.

Comparison	Antigen	Fold-difference (95% CI)	*p*-value	IgG naive (95% CI)	IgG exposed (95% CI)
F AlOH vs. BF Saline	FVO	1.00 (0.31 to 3.24)	1.000	16.9 (7.5 to 37.9)	16.8 (8.4 to 33.6)
F GLA-SE vs. BF Saline	FVO	1.88 (0.51 to 6.85)	0.573	31.5 (12.9 to 77.2)	16.8 (8.4 to 33.6)
F AlOH vs. BF GLA-SE	FVO	*0.26* (*0.08 to 0.86)*	*0.021*	16.9 (7.5 to 37.9)	64.8 (41.4 to 101.7)
F GLA-SE vs. BF GLA-SE	FVO	0.49 (0.13 to 1.80)	0.468	31.5 (12.9 to 77.2)	64.8 (41.4 to 101.7)
F AlOH vs. BF Saline	3D7	0.84 (0.25 to 2.77)	0.979	17.3 (7.3 to 41.2)	20.7 (9.7 to 44.1)
F GLA-SE vs. BF Saline	3D7	1.22 (0.33 to 4.58)	0.978	25.2 (12.6 to 50.4)	20.7 (9.7 to 44.1)
F AlOH vs. BF GLA-SE	3D7	*0.29* (*0.09 to 0.98)*	*0.046*	17.3 (7.3 to 41.2)	59.3 (38.2 to 92.1)
F GLA-SE vs. BF GLA-SE	3D7	0.43 (0.11 to 1.62)	0.337	25.2 (12.6 to 50.4)	59.3 (38.2 to 92.1)
F AlOH vs. BF Saline	HB3	1.00 (0.31 to 3.22)	1.000	18.5 (8.2 to 42.0)	18.6 (8.8 to 39.2)
F GLA-SE vs. BF Saline	HB3	1.43 (0.39 to 5.21)	0.884	26.5 (13.6 to 51.6)	18.6 (8.8 to 39.2)
F AlOH vs. BF GLA-SE	HB3	0.36 (0.11 to 1.17)	0.108	18.5 (8.2 to 42.0)	52.2 (32.6 to 83.5)
F GLA-SE vs. BF GLA-SE	HB3	0.51 (0.14 to 1.88)	0.523	26.5 (13.6 to 51.6)	52.2 (32.6 to 83.5)
F AlOH vs. BF Saline	CAMP	0.76 (0.23 to 2.57)	0.935	12.5 (5.5 to 28.3)	16.4 (7.5 to 35.8)
F GLA-SE vs. BF Saline	CAMP	1.46 (0.38 to 5.55)	0.876	23.9 (11.7 to 48.6)	16.4 (7.5 to 35.8)
F AlOH vs. BF GLA-SE	CAMP	0.30 (0.09 to 1.04)	0.061	12.5 (5.5 to 28.3)	41.1 (25.0 to 67.6)
F GLA-SE vs. BF GLA-SE	CAMP	0.58 (0.15 to 2.24)	0.711	23.9 (11.7 to 48.6)	41.1 (25.0 to 67.6)
F AlOH vs. BF Saline	DiCo1	0.62 (0.16 to 2.32)	0.767	19.6 (9.9 to 38.7)	31.8 (11.9 to 84.6)
F GLA-SE vs. BF Saline	DiCo1	1.19 (0.27 to 5.13)	0.990	37.7 (25.9 to 54.9)	31.8 (11.9 to 84.6)
F AlOH vs. BF GLA-SE	DiCo1	0.21 (0.06 to 0.81)	0.018	19.6 (9.9 to 38.7)	92.2 (47.6 to 178.7)
F GLA-SE vs. BF GLA-SE	DiCo1	0.41 (0.09 to 1.80)	0.386	37.7 (25.9 to 54.9)	92.2 (47.6 to 178.7)
F AlOH vs. BF Saline	DiCo2	0.54 (0.16 to 1.82)	0.542	24.5 (11.8 to 50.8)	45.1 (18.2 to 111.6)
F GLA-SE vs. BF Saline	DiCo2	1.42 (0.37 to 5.42)	0.897	64.2 (47.9 to 86.2)	45.1 (18.2 to 111.6)
F AlOH vs. BF GLA-SE	DiCo2	*0.15* (*0.04 to 0.51)*	*0.001*	24.5 (11.8 to 50.8)	162.4 (99.0 to 266.3)
F GLA-SE vs. BF GLA-SE	DiCo2	0.40 (0.10 to 1.53)	0.276	64.2 (47.9 to 86.2)	162.4 (99.0 to 266.3)
F AlOH vs. BF Saline	DiCo3	0.89 (0.27 to 2.96)	0.993	22.1 (11.2 to 43.7)	24.9 (9.7 to 64.4)
F GLA-SE vs. BF Saline	DiCo3	1.73 (0.46 to 6.57)	0.693	43.2 (31.0 to 60.4)	24.9 (9.7 to 64.4)
F AlOH vs. BF GLA-SE	DiCo3	*0.21* (*0.06 to 0.73)*	*0.008*	22.1 (11.2 to 43.7)	103.1 (67.4 to 157.7)
F GLA-SE vs. BF GLA-SE	DiCo3	0.42 (0.11 to 1.61)	0.328	43.2 (31.0 to 60.4)	103.1 (67.4 to 157.7)

Fold-difference and IgG values are expressed as geometric means with corresponding 95% confidence intervals for naive- and malaria-exposed volunteers. Fold-difference is expressed as the ratio between the naive and malaria-exposed geometric mean IgG levels. *p*-values are adjusted for multiple comparisons within antigen by Tukey’s post hoc test. Italics indicates statistically significant differences.

### Functional GIA levels to three laboratory strains in malaria-naive subjects

**Fig. 2:**
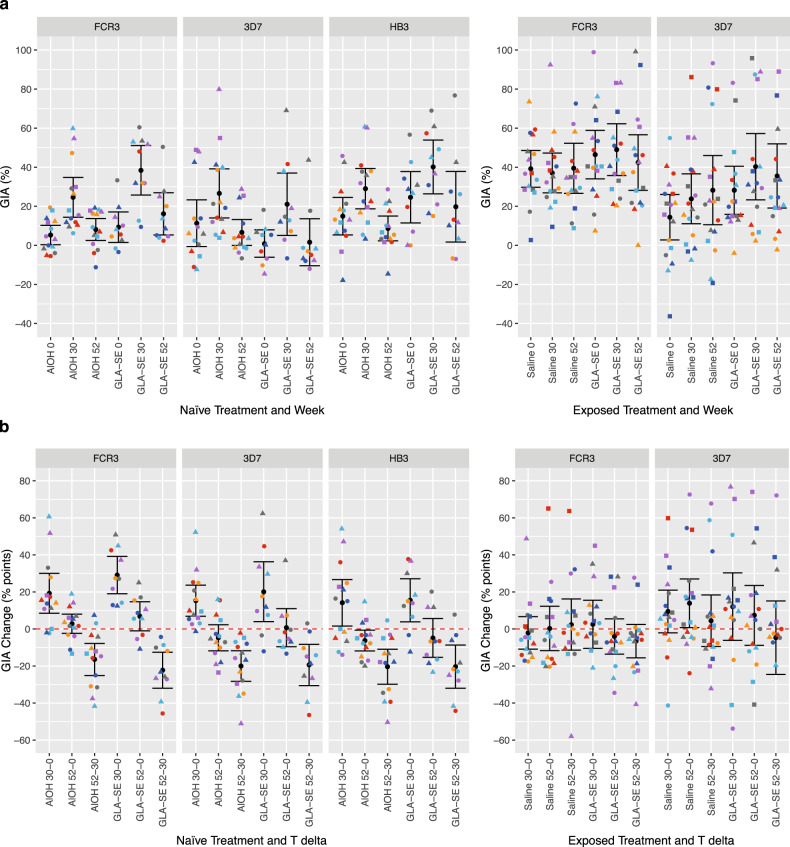
GIA levels and responses before vaccination, and four and 22 weeks after the third vaccination. **a** GIA levels at weeks zero, four and 22 weeks after the third vaccination and **b** GIA level changes following vaccination. Colours and shapes within treatment groups indicate individual subjects. Summary statistics (arithmetic means with 95% confidence intervals) are shown in every panel. The red dashed line in the GIA level change indicates no change in GIA level (post/pre-vaccination = 0).

To evaluate a broadening of the functional antibody response, we investigated growth inhibition of multiple lab-adapted malaria strains, using purified IgG from vaccinated individuals. We used two West-African strains (3D7 and FCR3) and a South-American strain (HB3). The number of amino acid differences in the AMA1 molecules of these variants are shown in Supplementary Table 1. GIA levels in the French cohort measured in samples obtained at weeks zero, 30 and 52 are shown in the left three panels of Fig. 2a.

Unexpectedly, FCR3, 3D7 and HB3 GIA levels at week zero differed from the expected value for malaria-naive subjects (viz. ±20%) for a number of subjects. To compensate for these differences in pre-vaccination GIA levels, the percentage-point difference between the three time points was calculated and statistically evaluated. The left three panels in Fig. 2b show the difference in GIA levels between the three time points. Four weeks after the third vaccination, GIA levels to the three laboratory strains increased by 14 to 29 percent points (all *p*-values < 0.02) as compared to week zero values. No significant differences in GIA levels were observed between the Alhydrogel® and GLA-SE groups (*p* = 0.45, LMM). Six months following the third vaccination GIA levels for the HB3 strain decreased significantly below baseline for the Alhydrogel® group −6.3 (−11.9 to −0.7, *p* = 0.031), whereas GIA levels for the other strains returned to baseline (Fig. 2a left three panels). All week 52 GIA levels were significantly lower than week 30 levels (all *p* < 0.0006).

### Functional GIA levels to 2 laboratory strains in malaria-exposed adults

GIA levels in the Burkina-Faso cohort in samples obtained at weeks zero, 30 and 52 are shown in the right two panels of Fig. 2a. Available amounts of purified IgG were limiting, therefore only FCR3 and 3D7 (two African isolates) GIA assays were performed. For the FCR3 strain GIA mean levels ranging between 37 and 49% were observed across treatment and time points and no significant changes in GIA levels were observed between the time points (Fig. 2a right panel). For the 3D7 strain GIA mean levels ranging between 14 and 40% were observed across treatment groups and time points (Fig. 2a right panel). With exception of the week 52 – day 0 difference, which was significantly positive in the Saline group with 13.9%-points (0.7 to 27.0, *p* = 0.04, paired *t*-test), GIA levels to the 3D7 strain remained unchanged over time (Fig. 2b right panel). Supplementary Fig. 2 (panel B) shows that there were no GIA level change differences within the treatment groups when comparing subjects with or without a symptomatic malaria episode.

### DiCo vaccination-induced GIA levels in malaria-naive adults are similar to GIA levels in placebo-vaccinated malaria-exposed adults

To confirm that AMA1-DiCo vaccination induces functional antibodies in malaria-naive subjects, we compared post vaccination GIA levels to those in malaria-exposed adults receiving placebo or GLA-SE adjuvanted AMA1-DiCo. Table 2 shows that GIA levels in the naive GLA-SE group are similar to those in the malaria-exposed Saline group. GIA levels in the naive Alhydrogel® group tended to be lower when compared to malaria-exposed subjects, but only achieved statistical significance for the naive Alhydrogel® with exposed GLA-SE group comparison.

**Table 2:** Comparison of Week 30 GIA levels in malaria-naive and malaria-exposed adults.

Ag	Comparison	Difference (95% CI)	*p*-value	GIA naive (95% CI)	GIA exposed (95% CI)
FCR3	F AlOH vs. BF Saline	−12.5 (−32.3 to 7.3)	0.348	24.6 (14.4 to 34.8)	37.1 (26.9 to 47.2)
FCR3	F GLA-SE vs. BF Saline	1.4 (−20.5 to 23.2)	0.998	38.4 (25.7 to 51.1)	37.1 (26.9 to 47.2)
FCR3	F AlOH vs. BF GLA-SE	*−24.4* (*−44.2 to −4.6)*	*0.01*	24.6 (14.4 to 34.8)	49.0 (35.7 to 62.3)
FCR3	F GLA-SE vs. BF GLA-SE	−10.6 (−32.4 to 11.3)	0.576	38.4 (25.7 to 51.1)	49.0 (35.7 to 62.3)
3D7	F AlOH vs. BF Saline	2.8 (−22.1 to 27.6)	0.991	26.6 (14.0 to 39.2)	23.8 (11.1 to 36.6)
3D7	F GLA-SE vs. BF Saline	−2.8 (−30.3 to 24.7)	0.993	21.0 (5.1 to 37.0)	23.8 (11.1 to 36.6)
3D7	F AlOH vs. BF GLA-SE	−13.7 (−38.9 to 11.6)	0.482	26.6 (14.0 to 39.2)	40.3 (23.3 to 57.2)
3D7	F GLA-SE vs. BF GLA-SE	−19.2 (−47.0 to 8.6)	0.268	21.0 (5.1 to 37.0)	40.3 (23.3 to 57.2)

Post vaccination percent-point differences in GIA levels with corresponding 95% confidence intervals for naive- and malaria-exposed volunteers. *p*-values are adjusted for multiple comparisons within antigen by Tukey’s post hoc test. Italics indicates statistically significant differences.

### IgG subclasses

Pre- and post-vaccination IgG1 levels and fold-increases to the three DiCo vaccine variants in malaria-naive adults are shown in Supplementary Fig. 3A. Pre-vaccination geometric mean IgG1 levels to the DiCo variants were about 1 AU/mL and increased 200-fold in the Alhydrogel® group and 400 to 800-fold in the GLA_SE group 4 weeks after the third vaccination. However, this difference between treatment groups in IgG1 fold-rise was not statistically significant. Post-vaccination IgG1 levels ranged between 939 and 10,000 AU/mL and tended to be about fourfold higher in the GLA-SE as compared to the Alhydrogel® group, but failed to achieve statistical significance (Supplementary Fig. 3A).

Pre- and post-vaccination IgG1 levels and fold-increases in malaria-exposed adults are shown in Supplementary Fig. 3B. Before vaccination, geometric mean IgG1 levels to the three DiCo variants were about 3000 to 4000 AU/mL. Four weeks following the third vaccination, IgG1 levels remained unchanged in the placebo group, whereas IgG1 levels in the GLA-SE group increased significantly by threefold (all *p* < 0.015). The post-vaccination geometric mean IgG1 levels to the DiCo variants were significantly higher (about fourfold) in the GLA-SE group as compared to the Saline group (all *p* < 0.007).

Pre- and post-vaccination IgG3 levels and post-vaccination fold-increases in malaria-naive adults are shown in Supplementary Fig. 4A. Pre-vaccination geometric IgG3 levels to the three DiCo variants were about 1 AU/mL and increased 8- to 20-fold 4 weeks after the third vaccination. The fold rises, however, did not differ between the treatment groups. Post vaccination IgG3 levels ranged between 9.2 and 36.8 AU/mL and did not differ significantly for the treatment groups. Pre- and post-vaccination IgG3 levels and post-vaccination fold-increases in malaria-exposed adults are shown in Supplementary Fig. 4B. Before vaccination IgG3 levels to the three DiCo variants were about 30 to 120 AU/mL. Four weeks following the third vaccination, IgG3 levels to the three DiCo variants remained unchanged in the placebo group and showed a slight, but non-significant, increase in the GLA-SE group. The resulting post-vaccination IgG3 levels did not differ significantly between the treatment groups.

### IgG avidity index

**Fig. 3:**
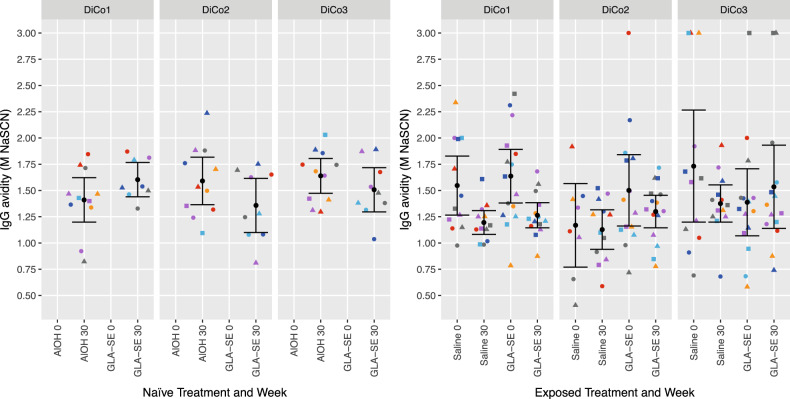
IgG Avidity Indices (AI) before vaccination and four weeks after the third vaccination to the AMA1 DiCo variants in malaria-naive and malaria-exposed adults. Individual IgG AI levels before vaccination and four weeks after the third are shown. Colours and shapes within treatment groups indicate individual subjects. Summary statistics (arithmetic means with 95% confidence intervals) are shown in every panel.

To assess the binding strength of the IgG antibodies, avidity indices (AI) were determined using a NaSCN elution ELISA. IgG AI for the two cohorts are shown in Fig. 3. Avidity indices before vaccination could not be determined for the French cohort, as levels for the AMA1-DiCo variants were too low to allow avidity quantification. Four weeks after the third vaccination, avidity indices were about 1.5 M NaSCN, irrespective of antigen or treatment (Fig. 3, panels 1 to 3). In malaria-exposed adults pre-vaccination avidity indices ranged between 1.2 and 1.75. Four weeks after the third vaccination the AI for DiCo1 and DiCo2 decreased significantly as compared to pre-vaccination in both treatment groups (all *p* < 0.008, paired *t*-test), whereas no statistically significant differences were observed for DiCo3. No statistically significant differences were observed between the treatment groups for the decrease in IgG avidity (Fig. 3).

### Competition ELISA

Competition ELISA was performed to confirm the cross-reactivity of the vaccine-induced responses.

**Fig. 4:**
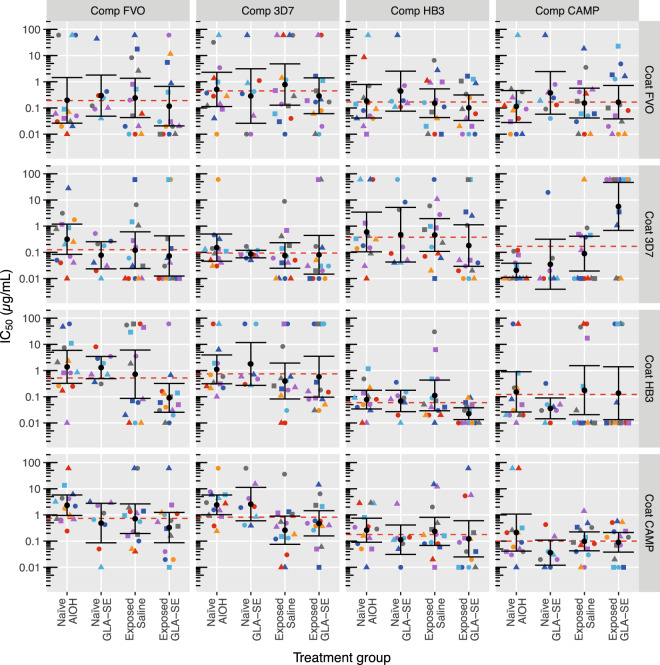
Competition ELISA IC_50_ values 4 weeks after the third vaccination for four natural AMA1 variants in malaria-naive and malaria-exposed adults. Individual IC_50_ values 4 weeks after the third vaccination are shown. Colours and shapes within treatment groups indicate individual subjects. Summary statistics (geometric means with 95% confidence intervals) are shown in every panel. The red dashed line indicates geometric mean IC_50_ values across the four treatment groups. Comp = Competitor antigen, Coat = coating antigen.

Figure 4 shows the amount of competitor required to reduce the level of IgG binding to the coated antigen by half (IC_50_). High IC_50_ values suggest limited antibody cross-reactivity, whereas low IC_50_ values suggest a high degree of cross-reactivity. When comparing IC_50_ values per competitor-coat pair, malaria-naive subjects did not differ from the saline malaria-exposed group and only two differences were found when comparing the malaria-naive subjects with the malaria-exposed GLA-SE group; with a lower IC_50_ for the FVO-HB3 competitor-coat pair and a higher IC_50_ for the CAMP-3D7 competitor-coat pair in the malaria-exposed GLA-SE group (Fig. 4). When comparing the geometric mean IC_50_ values for all treatment groups combined (red dashed lines in figure), homologous competitor-coat pairs (shown along the left to right diagonal in Fig. 4) show, as expected, low IC_50_ values: 0.19 (0.09 to 0.39), 0.09 (0.04 to 0.21), 0.06 (0.03 to 0.13) and 0.10 (0.06 to 0.18) µg/mL for FVO, 3D7, HB3 and CAMP, respectively. The amounts of heterologous competitor antigens for the FVO coat were significantly higher for 3D7 (2.32-fold, *p* = 0.033, LMM), and slightly lower (~0.87-fold, *p* > 0.74, LMM) for the HB3 and CAMP competitors. For the 3D7 coat antigen heterologous competitor levels were significantly higher (4.00-fold 1.34 to 11.92, *p* = 0.013, LMM) for the HB3 competitor and slightly higher (1.33-fold 0.45 to 3.95, *p* = 0.609 and 1.80-fold 0.61 to 5.36, *p* = 0.288, LMM) for FVO and CAMP, respectively. For the HB3 coat antigen heterologous competitor levels were significantly higher for the FVO and 3D7 competitors (8.79-fold 3.40 to 22.76, *p* < 0.001 and 12.53-fold 4.84 to 32.43, *p* < 0.001, LMM, respectively) and higher, albeit non-significantly for the CAMP competitor (2.06-fold 0.79 to 5.32, *p* = 0.136, LMM). For the CAMP coat antigen both FVO and 3D7 competitor levels were significantly higher when compared to the homologous competitor (7.41-fold 3.85 to 14.25, *p* < 0.001 and 8.17-fold 4.24 to 15.71, *p* < 0.001, LMM, respectively) and HB3 competitor levels tended to be slightly higher (1.78-fold 0.93 to 3.43, *p* = 0.083, LMM) than the homologous competitor.

### Cellular responses

The numbers of spot-forming cells following overnight stimulation with DiCo variants is shown in Supplementary Fig. 5. The number of spot-forming cells is expressed as the average of the three separate DiCo stimuli as reported by Sirima et al.^20^. In malaria-naive volunteers the Alhydrogel® group showed significant increases in the number of IL5 secreting cells following vaccinations, whereas no clear-cut IL5 responses were found in the GLA-SE group. In malaria-naive volunteers the number of IFNγ secreting cells was low (<5) and only few subjects responded following vaccination. Of note is the finding that post vaccination IFNγ responses in the GLA-SE group were low, which is not expected for a TLR4 agonist-containing adjuvant. In the malaria-exposed volunteers, numbers of IL5- and IFNγ-secreting cells increased following vaccination, unexpectedly this increase appeared to be more pronounced in the Saline group. Therefore, the observed increases are more likely due to ongoing malaria transmission than due to AMA1 DiCo vaccination.

Cytokine levels measured in most malaria-naive volunteers were low and, with few exceptions, did not change following vaccination (Supplementary Fig. 6A). In the malaria-exposed volunteers cytokine levels were detectable in a number of volunteers in the GLA-SE as well as in the Saline group. Therefore, the observed cytokine levels are likely due to ongoing malaria transmission, rather than due to vaccination (Supplementary Fig. 6B). Of note here is that the one volunteer in the Saline group (red square) with detectable levels for several cytokines experienced multiple (six) asymptomatic malaria episodes during the study period (Supplementary Table 2A).

## Discussion

The present study is an in-depth analysis of the outcomes of a clinical phase Ia/Ib combination trial with the malaria vaccine candidate PfAMA1 DiCo. The combination allows for a direct comparison between the malaria-naive and malaria-exposed volunteers, with the same batch of antigen and adjuvant. Primary outcome of the study showed that the vaccine was safe and well-tolerated, albeit that some quickly resolved adverse effects were observed, especially in the GLA-SE groups^20^.

The PfAMA1 DiCo vaccine was designed to overcome the polymorphisms present in the PfAMA1 protein with the objectives 1) to generate a vaccine that will induce a broad response to the multitude of strains found in the field, and 2) protecting target groups, e.g., travellers and infants from endemic areas, against severe disease.

The results presented here show that the first of the objectives has been achieved, in the sense that the data show that the French volunteers, as a proxy for the ultimate target group, have acquired IgG antibody levels against four natural AMA1 variants, differing between 17 to 29 amino acid residues, that are very similar in magnitude to non-vaccinated malaria-exposed adults. It should be noted, however, that immune responses in malaria-naive infants in endemic countries may differ from those in non-exposed adults.

The broadened response following DiCo vaccination is markedly different from the results obtained after vaccination with a single allele PfAMA1 protein that shows significantly lower responses against heterologous PfAMA1 proteins, where up to 50% lower levels have been found^9,14^ and IC_50_ values for heterologous competitors ranged between six and 88-fold^14^. The increased breadth of the humoral response is further confirmed by the competition ELISA experiments presented in Fig. 4. The overall profiles show broadened cross-reactivity of the antibodies between the different proteins, in particular for the FVO and 3D7 AMA1 variants where the homologous coat-competitor IC_50_ values were within a 2.3-fold range. For the HB3 and CAMP AMA1 variants as coating antigens it appears that the broadening separates in two groups where both HB3 and CAMP competitors were within a 2.1-fold range, whereas the FVO and 3D7 competitors were about 10-fold higher. In a previous study with a single variant AMA1-FVO vaccine IC_50_ values were 0.23 µg/mL (0.21 to 0.26), 1.47 µg/mL (1.08 to 2.02), 4.39 µg/mL (2.93 to 6.57) and 20.15 µg/mL (14.17 to 28.65) for FVO, HB3, CAMP and 3D7, respectively^14^, spanning an approximate 88-fold range. The ranges observed for heterologous competitors in the current study were decreased as compared to those obtained with a single variant vaccine and are indicative for a broadening of IgG responses.

Overall, the DiCo approach, using three different designed AMA1 sequences, here proves to be successful at inducing broadly reactive antibody responses. This is confirmed by the similarity in IgG levels for four natural AMA1 variants in the vaccinated malaria-naive volunteers with placebo-vaccinated malaria-exposed volunteers and the small IC_50_ value differences observed in the competition ELISA patterns. Other exploratory parameters did not reveal any differences between the French and the Burkinabe volunteers, other than a higher level of IgG3 antibodies in the Burkinabe volunteers. The decrease in post-vaccination IgG avidity for DiCo1 and DiCo2 as observed in the malaria-exposed volunteers was also found in the placebo group and, therefore is likely not due to vaccination, but rather due to ongoing malaria transmission.

Cellular responses as determined by ELISpot were modest in the malaria-naive volunteers, and low compared to previously obtained results^21^. The malaria-naive GLA-SE group unexpectedly showed low IFNγ responses, which may be explained by the relatively low dose of GLA (2.5 µg) used in the current study. Other fast-track studies for malaria vaccine candidates have shown that GLA-SE is able to elicit durable Th1-like immune responses^22^.

In the malaria-exposed volunteers, higher numbers of IFN-γ secreting cells were found following vaccination, but as this was also observed for the placebo group, it seems likely that these are the result of ongoing malaria transmission.

The second objective of the study was to induce potentially protective responses in the target population, in this case exemplified by the French volunteers, where GIA activity is entirely due to anti-AMA1 antibodies. In the GLA-SE adjuvanted group, the highest GIA level induced by vaccination was close to 40%, at an IgG concentration of 10 mg/mL. Calculations in a *P. knowlesi* non-human primate (NHP) model show that (AMA1-induced) IC_50_ values close to 3.4 mg/mL total IgG (50% inhibition at an IgG concentration of 3.4 mg/mL) controlled parasitaemia^23^, while Miura et al.^24^ and Payne et al.^12^ show that 100 µg/mL anti-AMA1-specific IgG, resulted in 50% inhibition, illustrating that the observed IgG concentrations (between 16.9 and 31.5 µg/mL) are in line with the observed GIA activities. This implies that vaccine-induced AMA1-specific antibody levels should be three- to fourfold higher to achieve potentially protective GIA levels^25^. The data presented here also show that neither Alhydrogel® nor GLA-SE are sufficiently potent to achieve these anti-AMA1 IgG levels, underscoring the need for a more potent adjuvant. Alternatively, or additionally, a different vaccination schedule may be used to achieve this goal. That alternative regimens can be successful is illustrated by a previous NHP *P. knowlesi* study^23^, in which the PkAMA1 protein was used with a potent adjuvant, combined with an extended vaccination schedule and inter-current infections, eventually resulting in the ability to control *Plasmodium knowlesi* blood-stage infection. Unfortunately, the development of highly potent, safe adjuvants is not a priority, hampered by the high costs associated with this. Without such adjuvant, potential vaccine candidates like PfAMA1 will not evolve to a stand-alone, efficacious malaria vaccine. Of note, care has to be taken to extrapolate NHP-data directly to the human situation. For example, the CHMI experiment of Payne et al.^12^ shows no correlation between GIA levels and parasite multiplication rates, while this was observed in the NHP study^23^, suggesting that the role of AMA1 in invasion may differ between *P. falciparum* and *P. knowlesi*.

GIA inhibition levels of the placebo-immunised Burkinabe volunteers were also found to be around 40% (Fig. 2b). This shows that vaccination with GLA-SE adjuvanted PfAMA1 DiCo induces the same level of inhibition as observed in a semi-immune adult living in an endemic area. At the same time, it raises the question as to why the Burkinabe adults are (expected to be) able to control parasitaemia, while the malaria-naive volunteers are not (expected to be) protected (as illustrated in Payne et al.^12^). One explanation may be the fact that malaria-exposed adults have antibodies to many different malaria antigens and that the observed protection is due to this antibody “cocktail”. Cellular responses could provide an alternative explanation, but we do not see a clear fingerprint for the involvement of cellular immunity, as the levels of cytokines are similarly low in both malaria-naive and malaria-exposed volunteers.

In the Burkinabe volunteers, only small increases were observed for the IgG levels against the natural variants, whereas the increases against the immunogens (PfAMA1 DiCo) were more pronounced. The GIA activity only marginally changed and it raises the question whether the AMA1 DiCo vaccination has had any influence on it. One possible explanation is provided by Miura et al., who show that GIA activity of anti-AMA1 antibodies is counteracted by antibodies against other (malaria) antigens^24^.

In summary, we have shown proof-of-concept for the “Diversity Covering” approach with the observation that the PfAMA1 DiCo vaccine induces broad range, cross-strain functional AMA1 antibodies in malaria-naive adults to levels similar as in adults in a malaria-endemic area.

Whether AMA1-alone vaccination can protect against clinical malaria is still debated.

While deletion of the ama1 gene does not yield viable (blood-stage) parasites^26^, a conditional knock-out study shows that 96% of (*Plasmodium berghei*) AMA1 is dispensable for parasite invasion of hepatocytes^27^. AMA1-vaccination studies with a highly potent adjuvant in malaria-naive subjects does not give rise to protection^12^, whereas a field study in malaria-exposed children showed that, although vaccination with AMA1 had no overall protective efficacy (17%), it appeared to have efficacy against parasites bearing a homologous AMA1 variant^28^. These paradoxical findings may be the result of the stringency of the CHMI, where the clinical end point is set at a much lower parasitaemia level than what is applied for the definition of clinical malaria^12,29^. Rhesus macaques are able to control *Plasmodium knowlesi* malaria infection after multiple PkAMA1 vaccinations (and intermediate challenge), in some cases after having experienced peak parasitaemias close to 2%^23,30^. Bearing all data on PfAMA1 in mind, it is evident that PfAMA1-DiCo will not be stand-alone malaria vaccine without a very potent and safe adjuvant, if at all. This does not exclude the possibility of AMA1 being a component of a multi-antigen (multi-stage) malaria vaccine.

With the proof of concept for the Diversity Covering approach in people, tackling the polymorphism issue for PfAMA1, further exploration of the combination of PfAMA1 DiCo with a potent adjuvant as a stand-alone vaccine or in combination with other vaccine candidates is warranted.

## Methods

### Fast-track clinical trial strategy

To accelerate early-stage vaccine development, a fast-track strategy was developed by EVI. This involves a staggered multi-centre phase Ia/Ib design for the first-in-human evaluation, which allows proceeding to the phase Ib trial after review of the safety data following the first dose in the French subjects. Both Alhydrogel® and GLA-SE-adjuvanted vaccines were tested in the French volunteers and following confirmation of the GLA-SE vaccine’s safety the Alhydrogel formulation was dropped, because of its low immunogenicity in previous trials^14,21,31^. An additional reason to drop the Alhydrogel® formulation was that it tends to induce Th2-biassed responses, whereas GLA-SE, containing a TLR4 agonist, is expected to induce Th1-biassed responses. The control group in the Ib phase received Saline.

### Participants

The results reported here apply to the per-protocol subjects as reported by Sirima et al.^20^. Participants were healthy males and non-pregnant females aged 20–45 years. The French subjects were recruited from the Paris region. The Burkinabe subjects were recruited from the Saponé Health District, approximately 50 km southwest of Ouagadougou, the capital of Burkina Faso. Malaria transmission is seasonal and is low during the dry season (November–May) and high during the rainy season (June–October). Subjects with symptoms, physical signs or laboratory values suggestive of systemic disorders; positive HIV, HBV and HCV tests were excluded. Additional exclusion criteria for European volunteers were: a history of malaria or travel in malaria-endemic areas within the past six months; positive serology for PfAMA1-DiCo; intention to travel to malaria-endemic countries during the trial. Written informed consent was obtained from each volunteer. The protocol was conducted in accordance with the Declaration of Helsinki and International Committee of Harmonisation Good Clinical Practice Guidelines and approved by the relevant ethics committees and regulatory authorities of France and Burkina Faso.

### Vaccines, vaccination and blood samples

The first vaccination was given in January 2014 in France and in July 2014 in Burkina Faso and the final visits were in March and July 2015, respectively. Each vaccine contained a total of 50 µg PfAMA1-DiCo variants (divided 1:1:1 for three DiCo’s) formulated with Alhydrogel® or GLA-SE (IDRI, Seattle) in a final volume of 0.5 mL. Vaccines were given intramuscularly in alternating arms on day 0, week 4 and week 26^20^. The malaria-naive cohort received either PfAMA1-DiCo-Alhydrogel® (*N* = 14) or PfAMA1-DiCo-GLA-SE (*N* = 10), whereas the malaria-exposed cohort received either placebo (Saline, *N* = 17) or PfAMA1-DiCo-GLA-SE (*N* = 16). Serum or plasma samples obtained on day 0 and week 30 were available for all analyses, while a week 52 sample was available for evaluation in GIA.

### ELISA (quantification, subclasses, competition and avidity)

Quantitative enzyme-linked immunosorbent assay (ELISA) was performed in duplicate on serum samples in 96 well half-area flat-bottomed microtitre plates (Greiner, Alphen a/d Rijn, The Netherlands), coated with 1 µg/mL purified PfAMA1 antigens according to published methods^14^. IgG levels to the three DiCo variants were reported previously^20^, and are here complemented with IgG levels to four natural PfAMA1 variants. The number of amino acid position differences for the seven AMA1 variants is shown in Supplementary Table 1. Goat anti-human IgG conjugated to alkaline phosphatase was used as conjugate for total IgG detection (Pierce, Rockford, IL). A serum pool from exposed African adults was included on each plate and antibody levels in the unknowns were calculated using a four-parameter fit. Anti-AMA1 variant IgG levels in the reference serum were quantified using SPR^20^.

Mouse monoclonal anti-human IgG subclass conjugated to horseradish peroxidase was used for IgG1 (Sanquin, Amsterdam The Netherlands, clone HP6188) or IgG3 detection (Sigma-Aldrich, Zwijndrecht, The Netherlands, clone HP-6050). A standard curve was included on each plate and antibody levels in the samples were calculated using a four-parameter fit. Titres are expressed as µg/mL for total IgG and as arbitrary units for IgG subclasses, where 1 AU/mL yields an OD of 1 over background.

The avidities of the antibodies were determined by sodium isothiocyanate (NaSCN) elution ELISA as previously described^14^. Briefly, microtitre plates were coated with PfAMA1 variant proteins as described above, following blocking, incubated with a pre-determined level (~2 AU) of IgG for 1 h. Plates were then washed and incubated with a NaSCN concentration range (from 0 to 3.0 M in 0.25 M steps) for 15 min. Plates were washed and developed with goat anti-human IgG alkaline phosphatase conjugate and substrate as previously described^14^. The avidity index (AI) is expressed as the concentration of NaSCN required for 50% dissociation of bound antibodies (relative to controls without NaSCN).

Competition ELISA was performed as previously described^18^. Dilutions that resulted in 2 AU were calculated for each serum sample and used in the subsequent antigen competition assay. The assay involved co-incubation of different allelic forms of PfAMA1, with test sera in plates coated with naturally occurring PfAMA1 variants, such that competition occurs between the added (competitor) antigens and the coated antigen. For the PfAMA1 variants the competitor antigens were diluted threefold over seven wells from 100 to 0.137 µg/mL with PfAMA1 from the FVO, 3D7, HB3 or CAMP variants. The fraction of IgG remaining bound with competitor added is divided by the IgG levels with no competitor added. The IC_50_ values (i.e., concentration of competitor required to displace half of the maximal binding) are calculated from the dose-response curves using non-linear regression.

### Growth inhibition assay (GIA)

Blood samples obtained at weeks zero, 30 and 52 were used for growth inhibition assays (GIA). The antibodies used in GIA were purified from serum or plasma (for French and Burkinabe subjects, respectively) on protein G columns (Immunopure Plus Pierce, St. Louis, MO, USA), exchanged into RPMI 1640 using Amicon Ultra-15 concentrators (30 kDa cutoff, Millipore, Ireland), filter-sterilised and stored at −20 °C until use. IgG concentrations were determined using a Nanodrop ND-1000 spectrophotometer (Nanodrop Technologies, Wilmington, DE, USA). *P. falciparum* strains FCR3, 3D7 and HB3 were cultured in vitro using standard culture techniques in an atmosphere of 5% CO_2_, 5% O_2_ and 90% N_2_. The GIA was performed as previously described, with minor modifications^9^. Briefly, the effect of purified IgG antibodies on in vitro parasite growth was evaluated at a single IgG (10 mg/mL). An IgG concentration of 10 mg/mL approximates the amount of IgG (7 to 17 mg/mL) found in undiluted human plasma^32^. Samples were run in triplicate using 96 well half-area flat-bottomed plates (Greiner, Alphen a/d Rijn, The Netherlands) with alanine-synchronised cultures of *P. falciparum* schizonts at an initial parasitaemia of 0.2–0.4%, a haematocrit of 2.0% and a final volume of 50 µL. After 40 to 42 h, cultures were re-suspended and transferred into 200 µL ice-cold PBS. The cultures were centrifuged, supernatants removed and the plates were frozen. Parasite growth was assessed by measuring parasite lactate dehydrogenase levels with the lactate diaphorase APAD substrate system, and plates were read at 655 nm after 30 min incubation in the dark. Parasite growth inhibition is calculated as follows: 100 × 1 − (OD schizont – OD background)/(OD_IgG – OD background). Parasite cultures without added IgG were used to define maximum parasite growth.

### Cytokine quantification

The cellular immune response was assessed in vitro by measuring production of the T-cell IL5 and IFNγ cytokines by ELISPOT following overnight stimulation with 10 µg/mL of the three DiCo variants on PBMC samples obtained at Day 0, Week 26, 30 and 52. A negative (unstimulated cells) and a positive control (cells stimulated with PMA-Ionomycin) were included for each subject, as previously described^33^. Numbers of spot-forming cells for IL5 and IFNγ were calculated as the average number of spots from the three separate DiCo stimuli as previously published^20^. Supernatants from the overnight stimulations for IL5 and IFNγ ELISpot assays as performed at the trial sites^20^ obtained at weeks 0, 26, 30 and 52 were pooled per time point and the three separate DiCo stimuli and assayed by a ProcartaPlex 10-plex assay for IFNγ, IL2, IL4, IL9, IL10, IL13, IL17A, IL21, TNFα and TNFβ (Thermo Fisher scientific, Breda, The Netherlands).

### Statistics

All statistical analyses were performed using Microsoft R-Open version 3.5.0^34^. Figures were prepared using the ggplot2 package in Microsoft R-open. Antibody levels (IgG and IgG subclasses) were log-transformed to obtain normality and are presented as geometric means with 95% confidence intervals throughout the manuscript. GIA levels and IgG avidity were approximately normally distributed and are presented as arithmetic means with 95% confidence intervals. The statistical significance of between group differences for pre-vaccination IgG levels was initially evaluated using one-way analysis of variance (ANOVA). Significant between group differences were further evaluated by Tukey’s Honest Significant Difference post hoc test, which applies a correction on the *p*-value for multiple comparisons and provides estimates with 95% confidence intervals (95% CI) for the between group comparisons. The statistical significance of post-vaccination between treatment differences was evaluated using linear mixed models (LMM) with antigen and treatment as fixed effects combined with antigen in subject as random effects.

### Reporting summary

Further information on research design is available in the Nature Research Reporting Summary linked to this article.

## Supplementary information

Supplementary Information

Reporting Summary

## Data Availability

The data that support the findings of this study are available from the corresponding author upon reasonable request.
