# Supplementary material for: Accelerated phase Ia/b evaluation of the malaria vaccine candidate PfAMA1 DiCo demonstrates broadening of humoral immune responses
Source: NPJ Vaccines. 2021 Apr 14;6:55. doi: 10.1038/s41541-021-00319-2 (PMC8046791; doi:10.1038/s41541-021-00319-2)
Supplement: Supplementary file 1 — Supplementary Information [file 41541_2021_319_MOESM1_ESM.pdf]

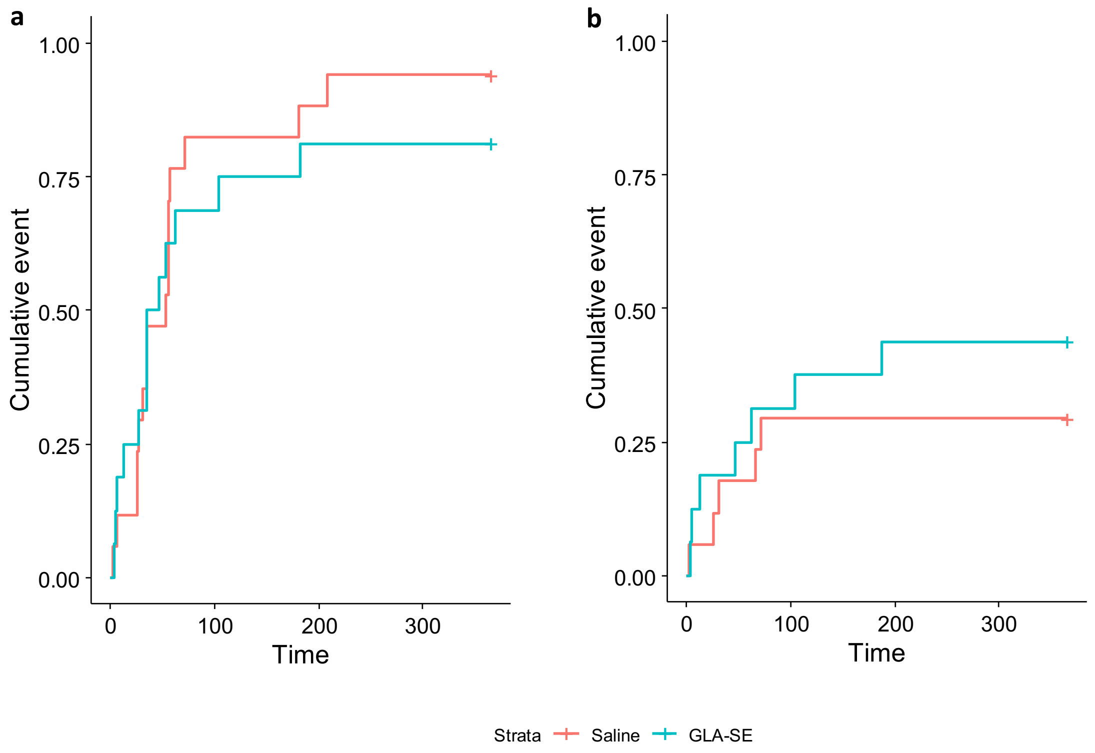

Supplementary Figure 1. Kaplan-Meier curves for the incidence of the first malaria episode in Burkinabe volunteers (either asymptomatic or symptomatic).

Panel a. time to first malaria episode (asymptomatic or symptomatic) and panel b. time to first symptomatic malaria episode.

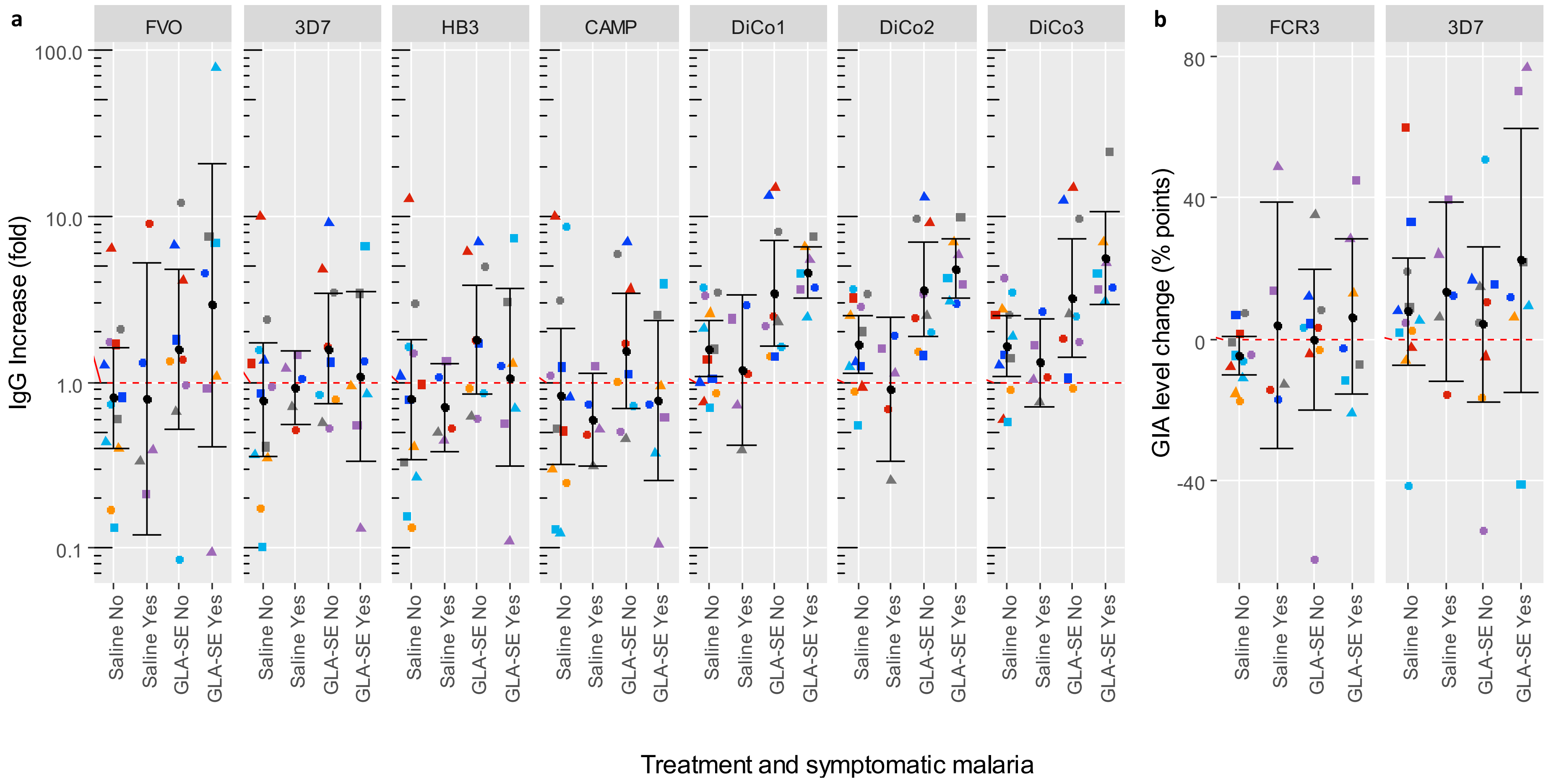

Supplementary Figure 2. Influence of symptomatic malaria episode on post-vaccination IgG and GIA responses in malaria-exposed subjects.

Panel a. IgG fold-increase and panel b. GIA level change. Colours and shapes within treatment groups indicate individual subjects. The red dashed line indicates no change in IgG (ratio post- / pre-vaccination = 1) or GIA level (post - pre = 0).

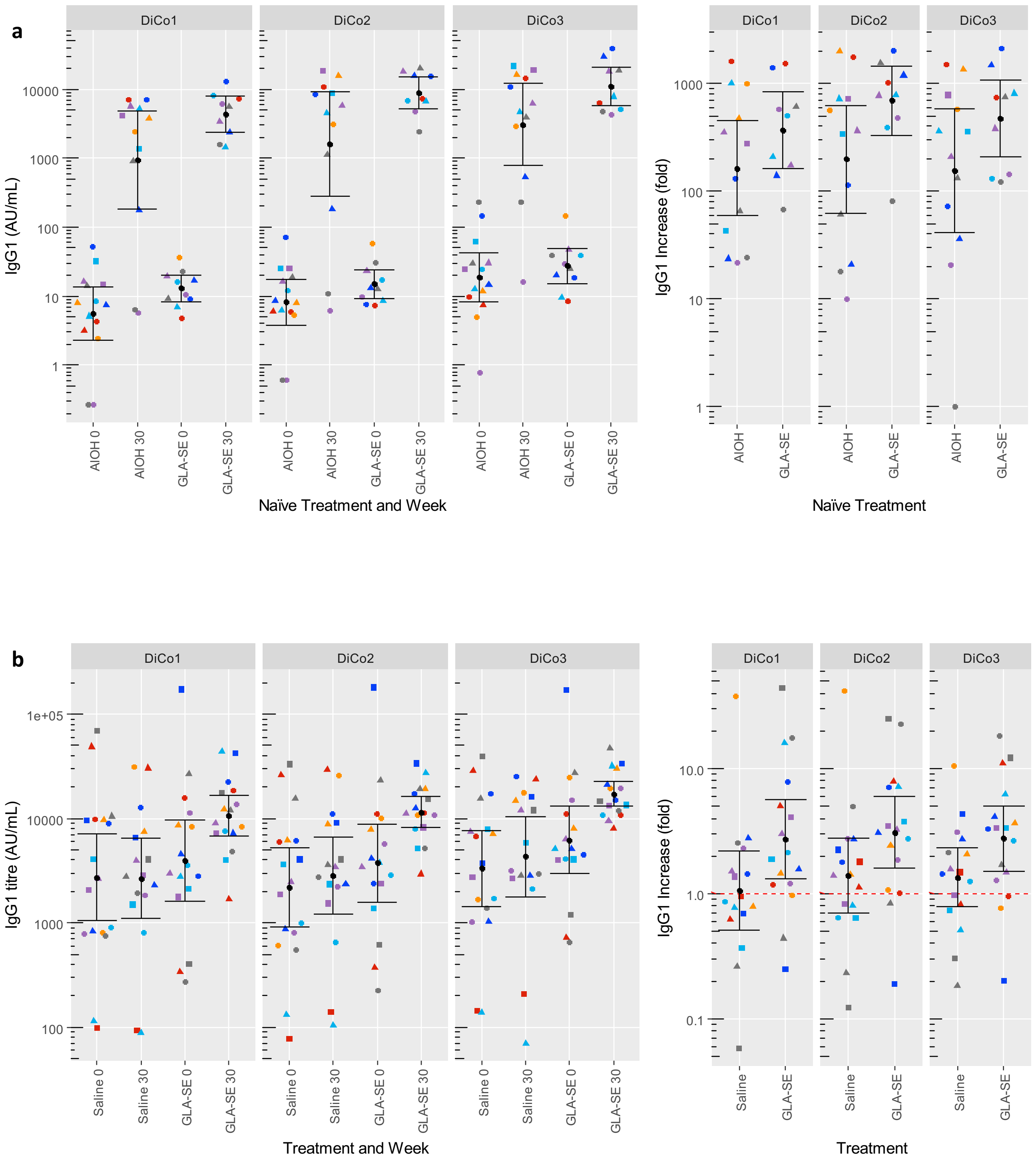

Supplementary figure 3. Pre and post-vaccination IgG1 levels and fold-increase to the vaccine antigens.

Panel a. Malaria-naïve adults and panel b. malaria-exposed adults. Individual IgG1 levels before and four weeks after the third vaccination and the IgG1 level ratios between week 30 and week 0 are shown. Colours and shapes within treatment groups indicate individual subjects. Summary statistics (geometric means with 95% confidence intervals) are shown in every panel. The red dashed line in the IgG1 increase indicates no change in IgG1 (ratio post- / pre-vaccination = 1).

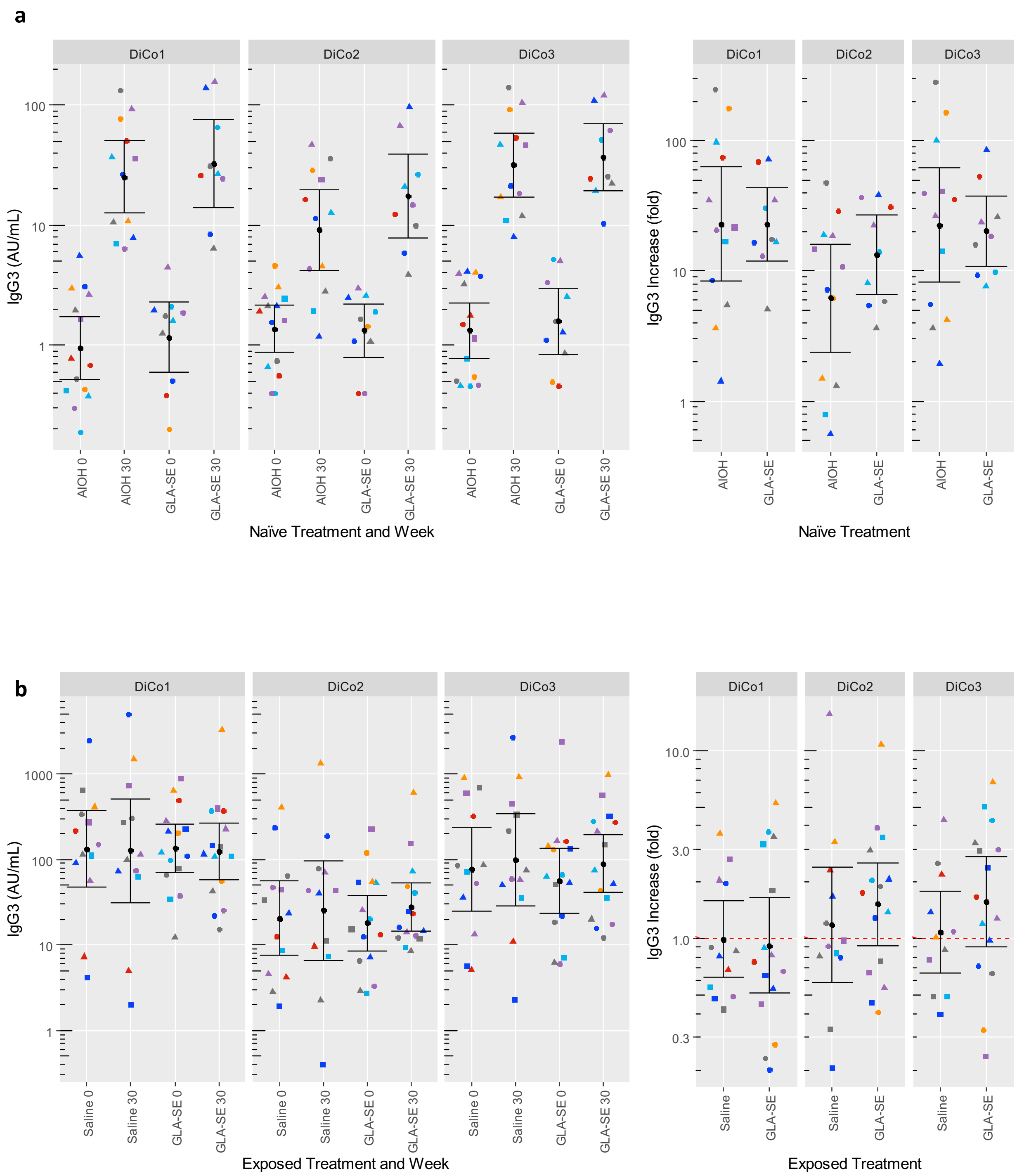

Supplementary figure 4. Pre and post-vaccination IgG3 levels and fold-increase to the vaccine antigens.

Panel a. Malaria-naïve adults and panel b. malaria-exposed adults. Individual IgG3 levels before and four weeks after the third vaccination and the IgG3 level ratios between week 30 and week 0 are shown. Colours and shapes within treatment groups indicate individual subjects. Summary statistics (geometric means with 95% confidence intervals) are shown in every panel. The red dashed line in the IgG3 increase indicates no change in IgG3 (ratio post- / pre-vaccination = 1).

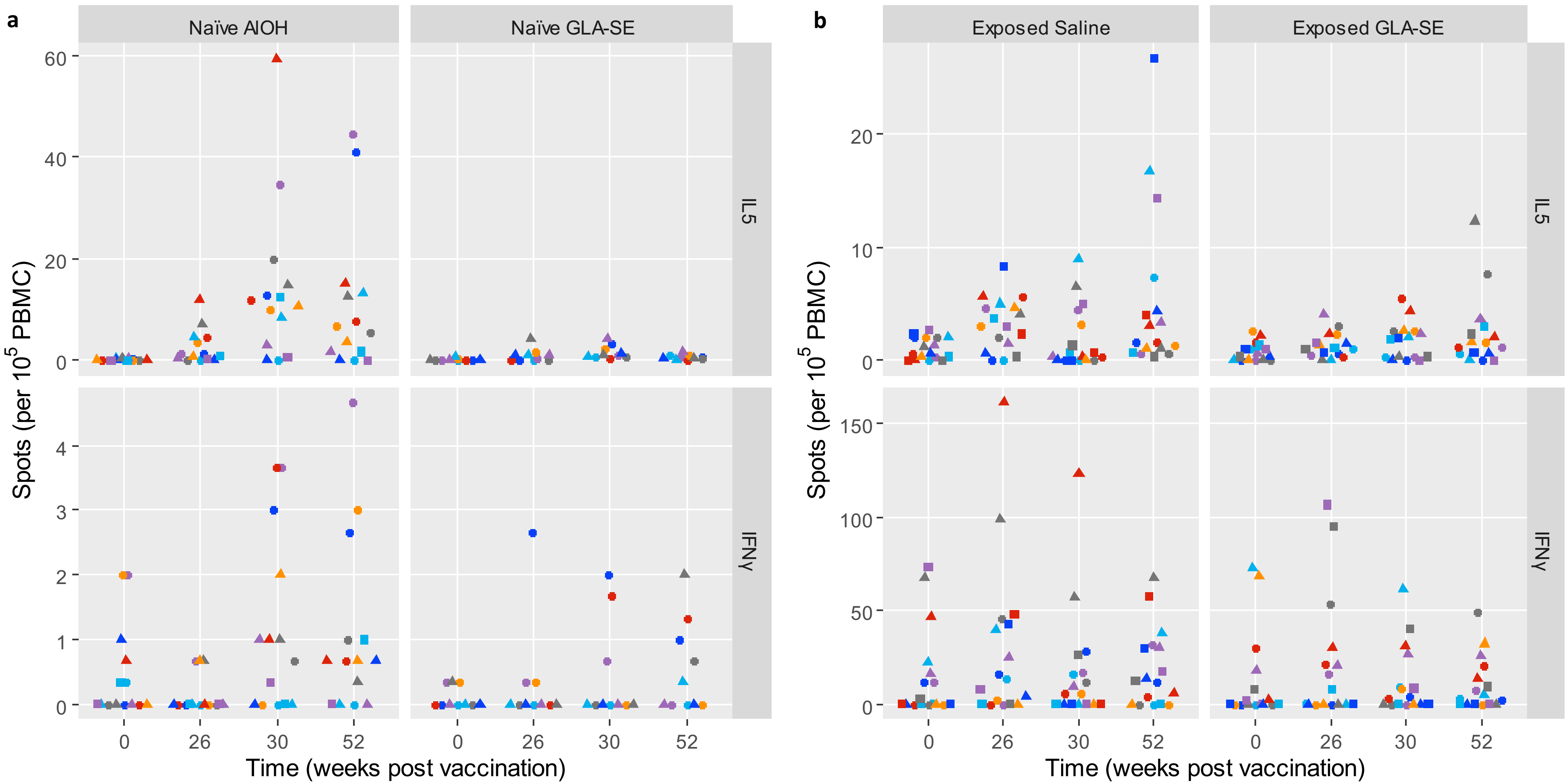

Supplementary Figure 5. Numbers of IL5 and IFNγ spot forming cells following stimulation with DiCo antigens.

Panel a. malaria-naïve adults and panel b. malaria-exposed adults. Colours and shapes within treatment groups indicate individual subjects.

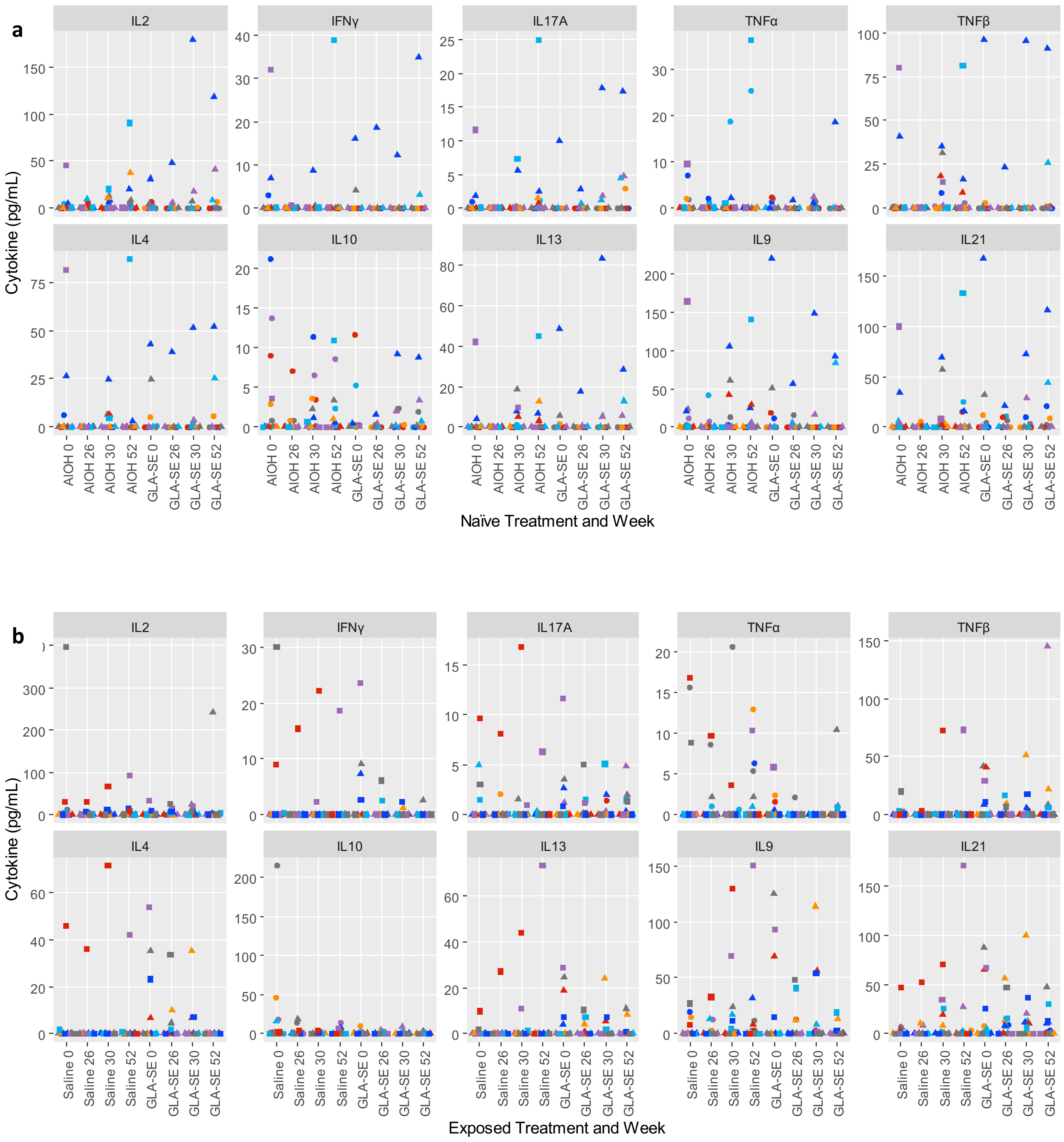

Supplementary Figure 6. Cytokine levels in ELISpot supernatants.

Panel a. malaria-naïve adults and panel b. malaria-exposed adults. Colours and shapes within treatment groups indicate individual subjects.

Supplementary Table 1. Number of amino acid position differences between the seven AMA1 variants

	FVO	3D7	HB3	CAMP	DiCo1	DiCo2	DiCo3
FVO	-	26	21	17	26	15	32
3D7	2-17-5-2	-	25	23	26	35	26
HB3	2-12-4-3	0-15-5-5	-	29	24	29	26
CAMP	3-9-3-2	3-12-6-2	3-16-5-5	-	28	20	40
DiCo1	0-16-5-5	2-9-10-5	2-11-9-2	3-12-6-7	-	23	18
DiCo2	0-5-7-3	2-20-10-5	2-14-7-6	3-10-6-1	0-15-2-6	-	34
DiCo3	0-17-8-7	2-10-7-7	2-12-8-4	3-17-11-9	0-11-5-2	0-19-7-8	-

Number of between AMA1 variant amino acid residue differences for the whole ectodomain (residues 25-545) are shown at the right of the diagonal. Differences per domain (pro, DI, DII, DIII) are shown at the left of the diagonal. Seven variant amino acids between DiCo and natural AMA1 variants (6 for HB3) are due to amino acid substitutions introduced to prevent protein glycosylation and cleavage. Substitutions to avoid glycosylation occur at residues 162 and 288 in domain I (residue 288 only for HB3), residues 373, 422 and 423 in domain II, and residue 499 in domain III. The substitution to avoid cleavage is at residue 376 (K to R) in domain II. The FVO and FCR3 laboratory strains differ by one residue (viz. residue 34 D to G) in the pro-domain.

Supplementary Table 2. Incidence of asymptomatic and symptomatic malaria

A. Malaria parasite densities per µL. W indicates week following immunisation.

Treatment	ID	W0	W1	W4	W5	W8	W26	W27	W30	W52	Symbol
**GLA-SE**	B-01					48					●
**GLA-SE**	B-08	6616				40					●
**GLA-SE**	B-28				40						●
**GLA-SE**	B-29		816					187			▲
**GLA-SE**	B-47				52						▲
**GLA-SE**	B-52		76						86		▲
**GLA-SE**	B-56			24							▲
**GLA-SE**	B-59									1041	▲
**GLA-SE**	B-61	123									■
**GLA-SE**	B-77						82			87	■
**GLA-SE**	B-79	552	174		139						■
**Saline**	B-07					128					●
**Saline**	B-10				324	3580					●
**Saline**	B-12			29873							●
**Saline**	B-16	413					475				●
**Saline**	B-21	140									●
**Saline**	B-25			135	108						▲
**Saline**	B-32		24	8	304						▲
**Saline**	B-34			812	72				32		▲
**Saline**	B-41					284	104	96			▲
**Saline**	B-43			1001							▲
**Saline**	B-48	1680	293		1580		380		108	55	■
**Saline**	B-49		1952								■
**Saline**	B-54					1282					■
**Saline**	B-65					221					■
**Saline**	B-67	176				333	194	261	76	52	■

B. Subjects without malaria episode(s)

Treatment	ID	Symbol
**GLA-SE**	B-02	●
**GLA-SE**	B-19	●
**GLA-SE**	B-26	●
**Saline**	B-21	●

C. Symptomatic malaria, day of first and / or second episode(s)

Treatment	ID	First	Second	Symbol
**GLA-SE**	B-08	5		●
**GLA-SE**	B-29	188		▲
**GLA-SE**	B-31	13		▲
**GLA-SE**	B-59	105		▲
**GLA-SE**	B-61	4		■
**GLA-SE**	B-72	63	222	■
**GLA-SE**	B-79	47		■
**Saline**	B-12	26	334	●
**Saline**	B-17	32		●
**Saline**	B-32	67		▲
**Saline**	B-35	72		▲
**Saline**	B-49	3		■
