# Supplementary material for: Accelerated phase Ia/b evaluation of the malaria vaccine candidate PfAMA1 DiCo demonstrates broadening of humoral immune responses
Source: NPJ Vaccines. 2021 Apr 14;6:55. doi: 10.1038/s41541-021-00319-2 (PMC8046791; doi:10.1038/s41541-021-00319-2)
Supplement: Supplementary file 2 — Reporting Summary [file 41541_2021_319_MOESM2_ESM.pdf]

nature research

Corresponding author(s): Bart W Faber
Last updated by author(s): Mar 15, 2021

Reporting Summary

Nature Research wishes to improve the reproducibility of the work that we publish. This form provides structure for consistency and transparency in reporting. For further information on Nature Research policies, see our Editorial Policies and the Editorial Policy Checklist.

Statistics

For all statistical analyses, confirm that the following items are present in the figure legend, table legend, main text, or Methods section.

n/a	Confirmed	
☐	☒	The exact sample size (*n*) for each experimental group/condition, given as a discrete number and unit of measurement
☐	☒	A statement on whether measurements were taken from distinct samples or whether the same sample was measured repeatedly
☐	☒	The statistical test(s) used AND whether they are one- or two-sided<br>*Only common tests should be described solely by name; describe more complex techniques in the Methods section.*
☐	☒	A description of all covariates tested
☐	☒	A description of any assumptions or corrections, such as tests of normality and adjustment for multiple comparisons
☐	☒	A full description of the statistical parameters including central tendency (e.g. means) or other basic estimates (e.g. regression coefficient) AND variation (e.g. standard deviation) or associated estimates of uncertainty (e.g. confidence intervals)
☐	☒	For null hypothesis testing, the test statistic (e.g. *F*, *t*, *r*) with confidence intervals, effect sizes, degrees of freedom and *P* value noted<br>*Give P values as exact values whenever suitable.*
☒	☐	For Bayesian analysis, information on the choice of priors and Markov chain Monte Carlo settings
☒	☐	For hierarchical and complex designs, identification of the appropriate level for tests and full reporting of outcomes
☒	☐	Estimates of effect sizes (e.g. Cohen's *d*, Pearson's *r*), indicating how they were calculated

Our web collection on statistics for biologists contains articles on many of the points above.

Software and code

Policy information about availability of computer code

Data collection	no software was used
Data analysis	Microsoft R-Open version 3.5.0, referenced in manuscript

For manuscripts utilizing custom algorithms or software that are central to the research but not yet described in published literature, software must be made available to editors and reviewers. We strongly encourage code deposition in a community repository (e.g. GitHub). See the Nature Research guidelines for submitting code & software for further information.

Data

Policy information about availability of data

All manuscripts must include a data availability statement. This statement should provide the following information, where applicable:

- Accession codes, unique identifiers, or web links for publicly available datasets
- A list of figures that have associated raw data
- A description of any restrictions on data availability

The data that support the findings of this study are available from the corresponding author upon reasonable request.

Field-specific reporting

Please select the one below that is the best fit for your research. If you are not sure, read the appropriate sections before making your selection.

☒ Life sciences ☐ Behavioural & social sciences ☐ Ecological, evolutionary & environmental sciences

For a reference copy of the document with all sections, see nature.com/documents/nr-reporting-summary-flat.pdf

Life sciences study design

All studies must disclose on these points even when the disclosure is negative.

Sample size	Human subjects were selected in France (30) and Burkina Faso (36). In the clinical Phase Ia/Ib between 30 and 40 individuals divided over 2 groups provide high enough statistical power to enable detection of significant effects. Especially in Africa, volunteers are more likely to drop out clinical studies, therefore a slightly larger initial group size was chosen.
Data exclusions	Of 30 French volunteers, the immunology of 25 was analysed. For the 36 Burkinabe, 33 were analysed. Reasons for exclusions are described in the article by Sirima et al: Sirima, S. B. et al. Safety and immunogenicity of a recombinant Plasmodium falciparum AMA1-DiCo malaria vaccine adjuvanted with GLA-SE or Alhydrogel(R) in European and African adults: A phase 1a/1b, randomized, double-blind multi-centre trial. Vaccine 35, 6218-6227, doi:10.1016/j.vaccine.2017.09.027 (2017).
Replication	Blood samples were analysed either in duplicate (IgG levels, Subclasses, avidity and competition ELISA) or in triplicate (Growth Inhibition assay and ELISpot).
Randomization	Randomization lists were generated with a 1:1 ratio using random block sizes of 2 and 4, independently by the trial statistician. See also Sirima, S. B. et al. Safety and immunogenicity of a recombinant Plasmodium falciparum AMA1-DiCo malaria vaccine adjuvanted with GLA-SE or Alhydrogel(R) in European and African adults: A phase 1a/1b, randomized, double-blind multi-centre trial. Vaccine 35, 6218-6227, doi:10.1016/j.vaccine.2017.09.027 (2017).
Blinding	This was a double-blinded study. See Sirima, S. B. et al. Safety and immunogenicity of a recombinant Plasmodium falciparum AMA1-DiCo malaria vaccine adjuvanted with GLA-SE or Alhydrogel(R) in European and African adults: A phase 1a/1b, randomized, double-blind multi-centre trial. Vaccine 35, 6218-6227, doi:10.1016/j.vaccine.2017.09.027 (2017).

Reporting for specific materials, systems and methods

We require information from authors about some types of materials, experimental systems and methods used in many studies. Here, indicate whether each material, system or method listed is relevant to your study. If you are not sure if a list item applies to your research, read the appropriate section before selecting a response.

Materials & experimental systems

n/a	Involved in the study	
☒	☐	Antibodies
☒	☐	Eukaryotic cell lines
☒	☐	Palaeontology and archaeology
☒	☐	Animals and other organisms
☐	☒	Human research participants
☐	☒	Clinical data
☒	☐	Dual use research of concern

Methods

n/a	Involved in the study	
☒	☐	ChIP-seq
☒	☐	Flow cytometry
☒	☐	MRI-based neuroimaging

Human research participants

Policy information about studies involving human research participants

Population characteristics	Al described in Sirima, S. B. et al. Safety and immunogenicity of a recombinant Plasmodium falciparum AMA1-DiCo malaria vaccine adjuvanted with GLA-SE or Alhydrogel(R) in European and African adults: A phase 1a/1b, randomized, double-blind multi-centre trial. Vaccine 35, 6218-6227, doi:10.1016/j.vaccine.2017.09.027 (2017).
Recruitment	for France: A database of healthy volunteers;<br>Posters of recruitment placed in different units of Cochin Hospital indicating the aim of the study, the rate of the compensation of participation and the phone contact of the CIC Cochin.<br>The CIC web site.<br><br>For Burkina Faso:<br>Meeting and exchange information with the authorities (community leaders, authorities of health)

	Meeting and exchange information with the community on the trial (obtain the assent of the community)<br>Selection during a public meeting (through a traditional lottery game) of a defined number of volunteers for possible participation in the trial.<br>These volunteers are invited to come to the clinical trial site for the next steps of the clinical trial procedures<br>Obtaining of the informed consent and screening for eligibility.
Ethics oversight	The protocol was conducted in accordance with the Declaration of Helsinki and International Committee of Harmonization Good Clinical Practice Guidelines and approved by the relevant ethics committees and regulatory authorities of France and Burkina Faso.<br>See Sirima, S. B. et al. Safety and immunogenicity of a recombinant Plasmodium falciparum AMA1-DiCo malaria vaccine adjuvanted with GLA-SE or Alhydrogel(R) in European and African adults: A phase 1a/1b, randomized, double-blind multi-centre trial. Vaccine 35, 6218-6227, doi:10.1016/j.vaccine.2017.09.027 (2017).

Note that full information on the approval of the study protocol must also be provided in the manuscript.

Clinical data

Policy information about clinical studies

All manuscripts should comply with the ICMJE guidelines for publication of clinical research and a completed CONSORT checklist must be included with all submissions.

Clinical trial registration	ClinicalTrials.gov NCT02014727
Study protocol	All can be found on the ClinicalTrials.gov website under the registration number NCT02014727, or in Sirima, S. B. et al. Safety and immunogenicity of a recombinant Plasmodium falciparum AMA1-DiCo malaria vaccine adjuvanted with GLA-SE or Alhydrogel(R) in European and African adults: A phase 1a/1b, randomized, double-blind multi-centre trial. Vaccine 35, 6218-6227, doi:10.1016/j.vaccine.2017.09.027 (2017). If questions remain, the corresponding author can be contacted.
Data collection	All can be found on the ClinicalTrials.gov website under the registration number NCT02014727, or in Sirima, S. B. et al. Safety and immunogenicity of a recombinant Plasmodium falciparum AMA1-DiCo malaria vaccine adjuvanted with GLA-SE or Alhydrogel(R) in European and African adults: A phase 1a/1b, randomized, double-blind multi-centre trial. Vaccine 35, 6218-6227, doi:10.1016/j.vaccine.2017.09.027 (2017). If questions remain, the corresponding author can be contacted.
Outcomes	All can be found on the ClinicalTrials.gov website under the registration number NCT02014727, or in Sirima, S. B. et al. Safety and immunogenicity of a recombinant Plasmodium falciparum AMA1-DiCo malaria vaccine adjuvanted with GLA-SE or Alhydrogel(R) in European and African adults: A phase 1a/1b, randomized, double-blind multi-centre trial. Vaccine 35, 6218-6227, doi:10.1016/j.vaccine.2017.09.027 (2017). If questions remain, the corresponding author can be contacted.
